# Single-shot laser pulse reconstruction based on self-phase modulated spectra measurements

**DOI:** 10.1038/srep33749

**Published:** 2016-09-20

**Authors:** Elena A. Anashkina, Vladislav N. Ginzburg, Anton A. Kochetkov, Ivan V. Yakovlev, Arkady V. Kim, Efim A. Khazanov

**Affiliations:** 1 Institute of Applied Physics of the Russian Academy of Sciences, 603950 Nizhny Novgorod, Russia

## Abstract

We report a method for ultrashort pulse reconstruction based only on the pulse spectrum and two self-phase modulated (SPM) spectra measured after pulse propagation through thin media with a Kerr nonlinearity. The advantage of this method is that it is a simple and very effective tool for characterization of complex signals. We have developed a new retrieval algorithm that was verified by reconstructing numerically generated fields, such as a complex electric field of double pulses and few-cycle pulses with noises, pedestals and dips down to zero spectral intensity, which is challenging for commonly used techniques. We have also demonstrated a single-shot implementation of the technique for the reconstruction of experimentally obtained pulses. This method can be used for high power laser systems operating in a single-shot mode in the optical, near- and mid-IR spectral ranges. The method is robust, low cost, stable to noise, does not require a priori information, and has no ambiguity related to time direction.

Nowadays, ultrashort optical pulse characterization is a quite advanced field of research having at its disposal simple autocorrelation trace measurements for retrieving envelope pulse profiles^1^,^2^ as well as refined interferometric and non-interferometric methods with direct and iteration reconstruction algorithms for full characterization of complex electric fields^2^. The most widely used techniques are SHG FROG (Second Harmonic Generation Frequency-Resolved Optical Gating)^3^,^4^, SPIDER (Spectral Phase Interferometry for Direct Electric-Field Reconstruction)^5^, and SHIAC (Second Harmonic Interferometric Autocorrelation)^6^. However, pulse reconstruction is not a trivial task because it requires consideration of an ambiguity in phase retrieval^2^,^3^. Moreover, since these methods are based on second harmonic generation, there also arise questions related to phase-matching bandwidth in the nonlinear crystal for ultra-broadband signals. A particularly acute problem is the retrieval of mid-infrared (mid-IR) pulses obtained, for example, by optical parametric chirped pulse amplification systems (OPCPA)^7^.

Another important and challenging problem is light field characterization of high power laser pulses interacting with matter. The advent of femtosecond petawatt (PW) class laser systems^8^,^9^ and the advance towards the 10-PW^10^,^11^ level makes full reconstruction of complex optical fields, particularly in the single-shot mode, an actual problem. As an example, reconstruction of compressed relativistically intense laser pulses in plasma-wakefield experiments when output spectra are highly modulated with dips down to zero spectral intensity is challenging for the SPIDER technique and needs prior information for field reconstruction^12^.

To overcome some of these problems, more complicated modifications and novel methods for pulse characterization are in constant progress, taking into account features of the measured pulses depending on a specific target. As an example, the autocorrelation techniques based on third harmonic generation^13^,^14^ and on six-wave mixing^15^ provide an opportunity to reveal asymmetry of the femtosecond laser pulses. Pulse contrast for ultra-high peak-power lasers may be measured by a single-shot cross-correlator^16^. Besides SHG FROG, there are many varieties of self-referenced FROG techniques for different beam geometries: polarization gate (PG), self-diffraction (SD), third-harmonic generation (THG), and non-self-referenced ones which use an additional laser beam such as XFROG (cross-FROG) and blind FROG^3^,^4^. There are also FROG modifications adapted to measure partially coherent pulses, even down to the attosecond timescale^3^,^17^, to determine not only the intensity and phase profiles of ultrashort pulses but also their absolute carrier-envelope phase values^18^, and to characterize mid-IR pulses based on four-wave mixing (FWM) in a gas medium^19^. Also available are SPIDER-modifications extending the applicability range, including the homodyne optical technique for SPIDER (HOT-SPIDER)^20^, the spatially encoded arrangement for SPIDER (SEA-SPIDER)^21^, and so on^2^, as well as other interferometric techniques, such as a very advanced method for phase and intensity retrieval of e-fields (VAMPARE)^22^, spatio-temporal amplitude-and-phase reconstruction by Fourier-transform of interference spectra of high-complex-beams (STARFISH)^23^, or measurement of electric field by interferometric spectral trace observation (MEFISTO)^24^.

Recently, a thin film compressor was proposed which may open the door to the Zeptosecond-Exawatt physics^25^,^26^. It is based, in particular, on controllable high intensity laser pulse spectrum modification with subsequent pulse compression to single cycle duration. The influence of input pulse spectral phase on self-phase modifications was also studied^27^. Here, we report a robust method of full reconstruction of complex optical field based only on the fundamental spectrum (input pulse spectrum) and two SPM spectra measurements after thin media with Kerr nonlinearity. This method is very simple, can be applied for single-shot pulse characterization in the optical, near- and mid-IR spectral ranges, including very complex signals such as few-cycle pulses with complicated spectral and temporal structures. It should be noted that reconstruction of a one-dimensional spectral phase based on the fundamental and one SPM-spectrum was proposed earlier^28^,^29^. That method was also extended to time resolution of the polarization state of ultrashort light pulses^30^. However, the experimental data do not uniquely specify unknown spectral phase, so the field cannot be determined unambiguously without additional prior knowledge of pulse shape. The method also requires knowledge of the exact value of the B-integral. We assume that this method has not been widespread for the above reasons. The method we propose allows using a new retrieval algorithm for minimizing errors, which makes it a very effective tool free of the mentioned shortcomings. We believe that the method will find wide application because of its simplicity, low cost, stability to noise, absence of ambiguity related to time direction and dips down to zero spectral intensity.

## Results

### Description of the method

The proposed method is based on measuring only three spectra, the first of which is the fundamental *I*_*0*_(*ω*), the second *I*_*1*_(*ω*) and the third *I*_*2*_(*ω*) are after thin Kerr media with *B*-integrals differing twice. The *B*-integral is defined as


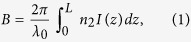


where *I*(z) is the optical intensity along the beam axis, *z* is the coordinate along the beam direction, *L* is the thickness of Kerr medium, *λ*_*0*_ is central wavelength, and *n*_*2*_ is the nonlinear index quantifying the Kerr nonlinearity. It is important to note that the method does not require a priori knowledge of numerical value of the *B*-integral. Contrariwise, it gives an estimate of the B-integral.

The electric field 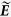 of a linearly polarized pulse propagating along the *z*-axis in Cartesian coordinates can be written in the slowly varying envelope approximation in the form


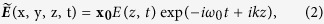


where *E* is complex amplitude, **x**_**0**_ is unit vector, *ω*_*0*_is central frequency, *t* is time, and *k* is propagation constant. The Fourier transformed field *E*(z, *ω*) is given by


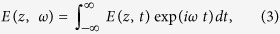


where *ω* is the angular frequency counted from the central frequency *ω*_*0*_. The spectral intensity is *I*(*z*, *ω*) = |*E*(*z*, *ω*)|^2^. We employ a fast Fourier transform (FFT) to re-calculate functions from the time to the frequency domain and an inverse FFT (IFFT) for re-calculation from the frequency to the time domain.

**Figure 1:**
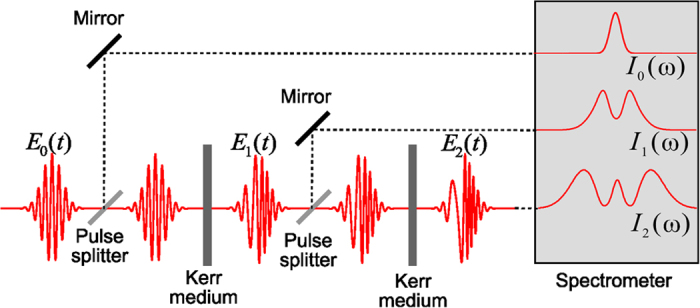
Schematic diagram of the proposed method.

The schematic of the method proposed is shown in Fig. 1.

Let us define *E*_*0*_(*t*) as a dimensionless complex amplitude of the measured pulse normalized to its maximum value, *E*_*1*_(*t*) as a dimensionless amplitude after the first thin layer of Kerr medium, and *E*_*2*_(*t*) as an amplitude after the second layer. One should use a thin medium neglecting other linear and nonlinear effects, such as dispersion, optical loss (except Fresnel reflection), diffraction, and nonlinearities except Kerr nonlinearity. For TW and PW class laser systems in the optical and near-IR range, it is possible to use sub-mm “plastic” films proposed by Mourou^25^, which could be amorphous polymer thermoplastics, triacetate of cellulose, polyester, or other elements as long as they are transparent to the wavelength under study, robust, flexible, and exhibit uniform thickness. The recent study^26^ has confirmed the applicability of polyethylene terephthalate films due to a large value of cubic nonlinearity, insignificant phase aberrations, negligibly small depolarization and losses, stability to high intensity, and low cost. Thin glass plates may be used as well. It is very easy to estimate the ratio of the first and the second films taking into account transmittance of the pulse splitter and Fresnel reflection of the plastic film. If one uses a pulse splitter after the first film with a small reflection coefficient (of about a few percent or lower), the second Kerr medium may be identical to the first one, or it is possible to use only one film and a system of mirrors to send the pulse repeatedly through it to obtain field *E*_*2*_(*t*). We will demonstrate by numerical examples that pulses can be retrieved reasonably well even if the *B*-integral difference is not exactly a factor of two.

The dimensionless fields after the thin films are related by^31^


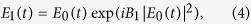



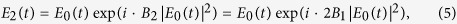


where *B*_*1*_ and *B*_*2*_ are the *B*-integrals after the first and the second Kerr layers. Let us define


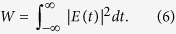


Further, the integrals of three spectra *I*_*0*_(*ω*), *I*_*1*_(*ω*) and *I*_*2*_(*ω*) are equated to each other and Parseval’s theorem is used:


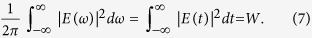


The spectral intensity of the reconstructed pulse *I*_*0*_(*ω*) is taken from the experiment and the spectral phase *φ*(*ω*) is chosen to minimize the difference between two measured SPM-spectra and the corresponding simulation. The error is defined by


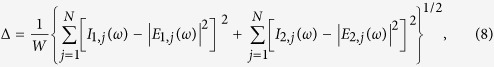


where *N* is the number of points in frequency domains.

### Numerical algorithm

**Figure 2:**
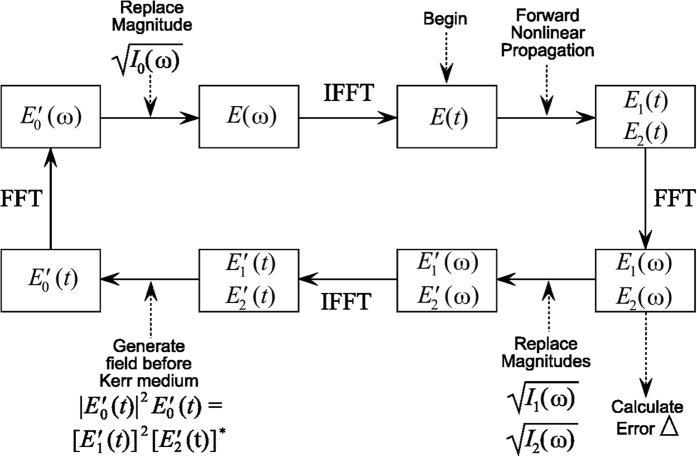
Schematic diagram of the algorithm developed using fundamental and two SPM-spectra with B-integrals differing twice.

We have developed a numerical algorithm to minimize the error defined by Eq. (8). It is based on the Gerchberg–Saxton algorithm that is an iterative algorithm for retrieving the phase of a pair of light distributions (or any other mathematically valid distribution) related via a propagating function, if their intensities at their respective optical planes are known^32^. The generalized numerical procedure for our case depicted in Fig. 2 is as follows.

Starting with the initial complex amplitude *E*(*t*), the nonlinear propagation is calculated by formulas (4) and (5), where *E*(*t*) is used instead of *E*_*0*_(*t*). This procedure yields *E*_*1*_(*t*) and *E*_*2*_(*t*). After FFT of *E*_*1*_(*t*) and *E*_*2*_(*t*) resulting in *E*_*1*_(*ω*) and *E*_*2*_(*ω*), their spectral phases remain unchanged but their magnitudes are replaced by the experimentally measured square roots from SPM-spectra, and IFFT are executed. The resulting complex amplitudes are *E*_*1*_′(*t*) and *E*_*2*_′(*t*). Further, we generate a new field *E*_*0*_′(*t*) before Kerr medium using the relation that is correct for a true pulse with (4) and (5) taken into account:


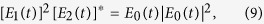


where the asterisk means complex conjugation. Instead of back propagation used in the algorithm with one measured SPM–spectra^28^,^29^, we take


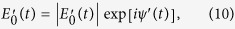


where 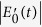 and *ψ*′(*t*) are found from


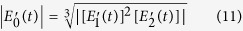



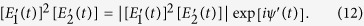


Recently, a Gerchberg–Saxton-like algorithm for temporal phase reconstruction has been demonstrated by sequentially applying multi-step intensity-only measurements^33^. But we use multi-SPM-spectra information in parallel and generate a new sought-for field at each iteration without backward nonlinear propagation. After that, FFT is applied and a standard procedure with replacing the spectral magnitude of 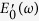 by square roots from the fundamental spectrum and IFFT of the resulting *E*(*ω*) are executed to complete the cycle with new *E*(*t*).

The error is estimated at each iteration. Two minimum values of Δ and the corresponding *E*(*t*) are saved. Let us define them as Δ_*min*_, Δ_*min-1*_ and *E*_*min*_(*t*), *E*_*min-1*_(*t*). To improve convergence, elements of genetic algorithm are used: (1) crossover, when *E*(*t*) is replaced by 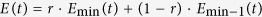, where *r* is a random value between 0 and 1, and (2) mutation, when the phase of *E*(*t*) is modified. These procedures are used if the error does not decrease on the average for a large enough number of iterations (usually of about 100).

The algorithm requires setting *B*-integrals which is not well-known as a rule. We propose to run the algorithm for various (*B*_*1*_*·W*), where *W* is defined by Eq. (6), and select the value leading to minimum Δ_*min*_. It will be demonstrated further with simulated examples that the minimum of the function Δ_*min*_(*B*_*1*_*·W*) is sharp enough and corresponds to *B*_*1*_ close to the original value. Besides, the difference up to several tens percent in *B*_*1*_*·W* usually does not lead to dramatic changes in retrieved pulse.

### Numerical examples

**Figure 3:**
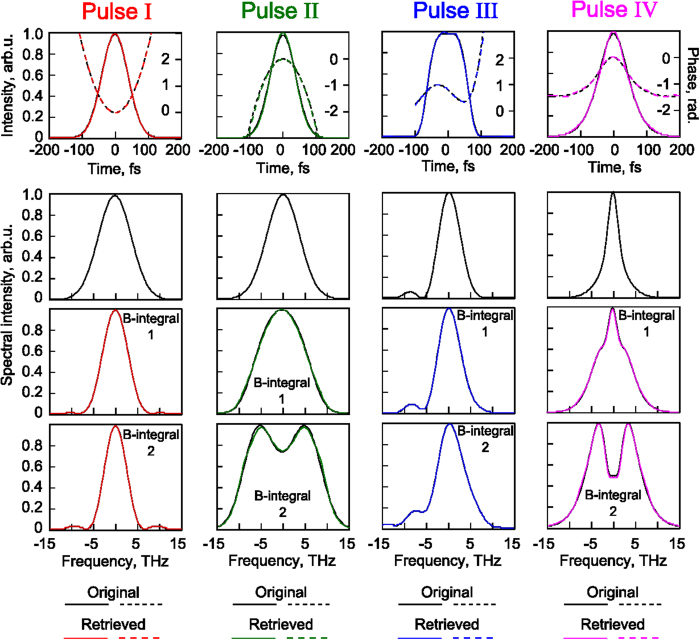
Reconstruction of test pulses. Intensity profiles of pulses (left axes, solid lines) and their phases (right axes, dotted lines) in the time domain: original (black) and retrieved (color) with the proposed method (1st horizontal row), normalized fundamental spectrum (2nd row) and normalized SPM spectra for B-integrals differing in twice (3rd and 4th rows, respectively).

First of all, the algorithm has been verified by using test signals such as Gaussian, Super-Gaussian, sech-form, and so on with polynomial and arbitrary phases. In all cases, it demonstrates very good results; in Fig. 3 we present some particular examples such as (pulse I)


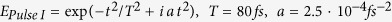


is Gaussian pulse with positive linear chirp, (pulse II)


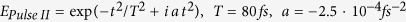


is Gaussian pulse with negative linear chirp, (pulse III)


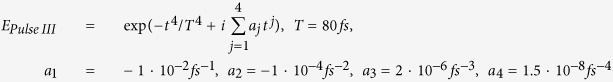


is super-Gaussian pulse with polynomial phase, and (pulse IV)


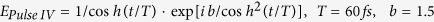


is phase modulated sech-form pulse. The corresponding intensity profiles and phases of the signals are shown in the first row of Fig. 3. The algorithm is running for the fundamental (the second row) and two SPM spectra with the B-integrals of 1 and 2 differing twice; see the third and fourth rows. One can see perfect agreement between the reconstructed and the original SPM spectra as well between intensities and phases in the time domain.

We have also analyzed pulses with perturbations such as noises, pedestals and B-integrals differing not exactly twice. For perturbations smaller than ~10%, retrieval pulses are usually very similar to the original ones, but for perturbations up to 20–30% the reconstruction also gives reasonable results. We have obtained almost perfect results without perturbations.

Next we consider in more details two examples, which are challenging for many widely used techniques. They are a double pulse and few-cycle pulse with noises, pedestals and dips down to zero spectral intensity. A comparison with characterization using the SHG-FROG method is also provided.

**Figure 4:**
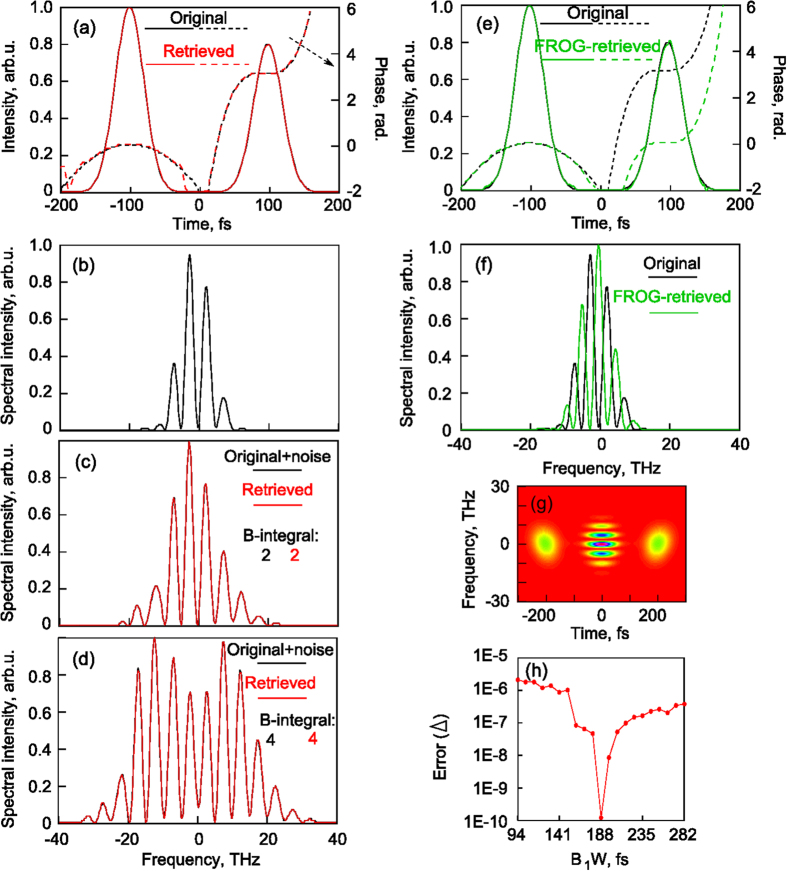
Reconstruction of double pulse. Its intensity profiles and phases in the time domain: original and retrieved with the proposed method (**a**), normalized fundamental spectrum (**b**) and normalized SPM spectra for B-integrals differing twice (**c**,**d**). (**h**) Error for different B_1_W. Original and reconstructed intensity profiles with FROG-method in the time (**e**) and frequency (**f**) domains. (**g**) FROG-trace.

The test concerning the double-pulse is not a trivial task for many methods, such as the commonly used FROG techniques^3^. But it should be noted that blind FROG^3^ or VAMPIRE^22^ can provide correct reconstructions even when there are extended areas of zero intensities between peaks in the temporal or spectral domains. The tested intensity profile and phase are plotted in Fig. 4(a). Its fundamental spectrum and SPM spectra calculated for B-integrals of 2 and 4 are shown in Fig. 4(b–d). We use the developed algorithm for *B*_*1*_*·W* varying in the range of 94–282 fs and obtain a minimal error Δ defined by Eq. (8) for *B*_*1*_*·W* = 188 fs (see Fig. 4(h)), providing a very good agreement between the tested and retrieved pulse and its SPM spectra shown in Fig. 4. The original and retrieved curves are almost identical without perturbations, and the obtained *B*_*1*_ is exactly 2. For this numerical example the FROG-method may not reconstruct relative phase between separated pulses and gives incorrect spectrum due to interference. The reconstructed intensity profile in the time and frequency domains in comparison with the original ones are shown in Fig. 4(e,f), respectively. The numerically generated SHG-FROG-trace^3^ for the original signal is given in Fig. 4(g). As the temporal separation between pulses determines the period of spectral fringes, the maximal delay that can be characterized using the proposed technique depends on the spectrometer resolution. The spectral resolution must be high enough to register these fringes, otherwise the method fails.

**Figure 5:**
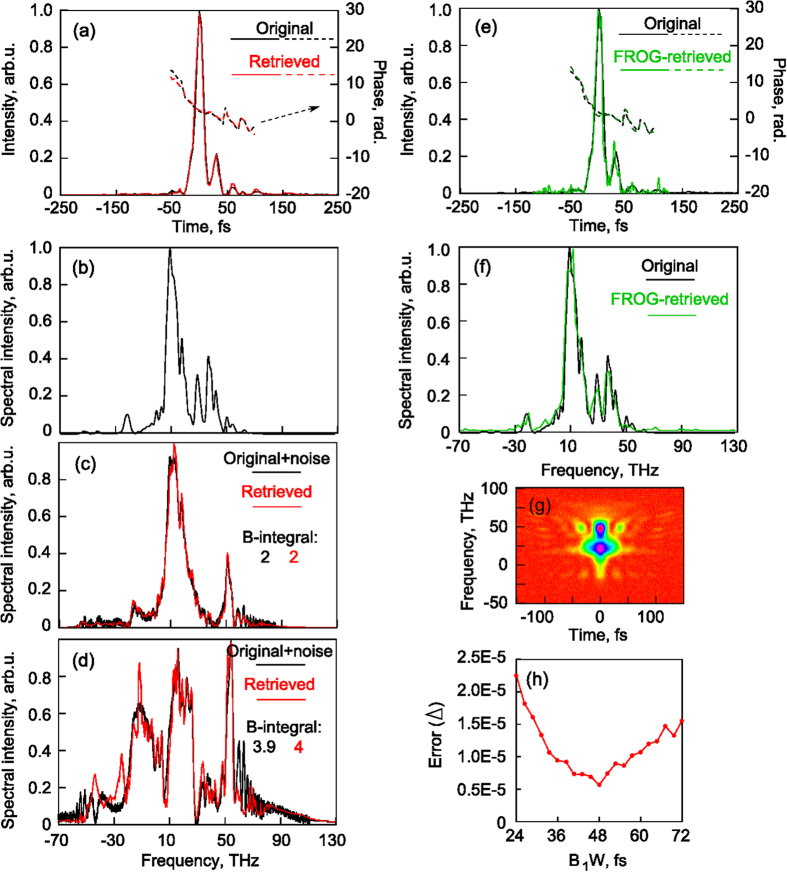
Numerical example of a few-cycle pulse. Its intensity profiles and phases in the time domain: original and retrieved with proposed method (**a**), normalized fundamental spectrum (**b**) and normalized SPM spectra for B-integrals differing about twice (**c**,**d**). (**h**) Error for different B_1_W. Original and reconstructed intensity profile with FROG-method in the time (**e**) and frequency (**f**) domains. (**g**) FROG trace.

The next example concerns few-cycle pulses obtained in our previous study on compression due to wakefield excitation in the relativistic regime using SPIDER^12^. The tested signal is shown in Fig. 5(a). It should be noted that the application of SPIDER was challenging as the spectrum was highly modulated and ultrabroadband (see Fig. 5(c)). Here we take the retrieved field and simulate the spectra after propagation through dispersionless Kerr media with B-integrals of 2 and 3.9. The corresponding results with added random noise of 5% from the maximum spectral intensities are plotted in Fig. 5(d,e). After that, we employ the developed algorithm by varying *B*_*1*_*·W* from 24 to 72 fs. The calculated errors Δ are presented in Fig. 5(b). The reconstructed intensity profile in the time domain and SPM spectra for *B*_*1*_*·W* = 48 fs which give the minimal error are given in Fig. 5(a,d,e) corresponding to *B*_*1*_ = 2 and *B*_*2*_ = 4. This example demonstrates a very good applicability of the proposed method and algorithm for few-cycle pulses even with pedestal and noise for unknown *B*-integrals differing not exactly twice. We have also obtained similar results for the reconstructed intensity profile with the FROG-method demonstrated in Fig. 5(e,f) in the time and frequency domains. However, due to the ambiguity related to time direction for the FROG-method, it is impossible to determine the leading and trailing pulse edges without additional measurements, but this may be very important, for example, in experiments where high-intensity few-cycle pulses interact with matter^12^.

## Experimental Results

**Figure 6:**
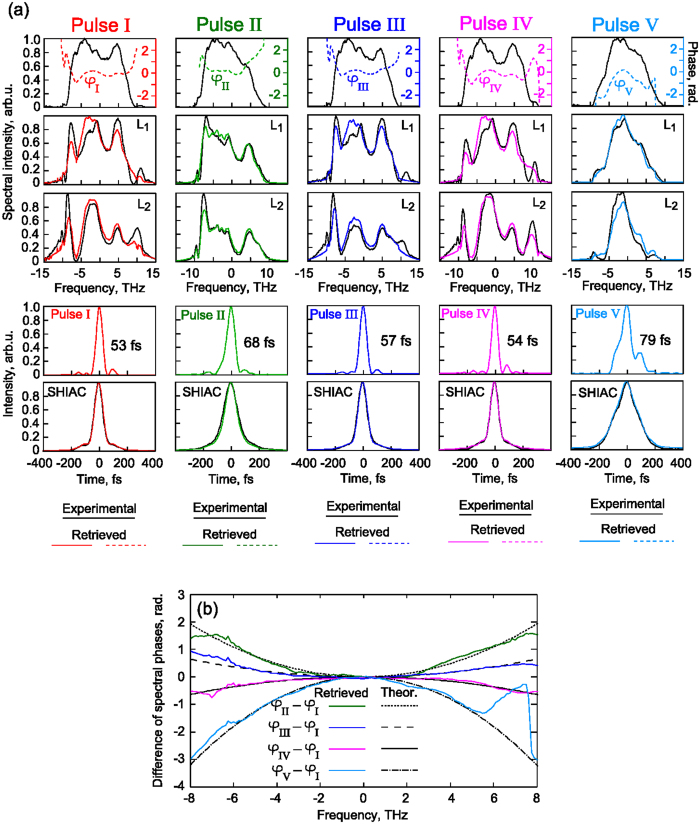
(**a**) Experimental results for 5 pulses obtained with different dispersive compressor settings: each column corresponds to a single-shot pulse. The 1st horizontal row contains measured fundamental spectra (left black axes) and retrieved spectral phases (right color axes), the 2nd and 3rd rows contain SPM spectra after Kerr media of 0.75 mm and 1.5 mm respectively. Here the zero group velocity dispersion frequency corresponds to a wavelength of 919 nm. The 4th row displays reconstructed intensity profiles, and the 5th row shows measured SHIAC-functions and numerically generated for reconstructed pulses from the 4^th^ row. (**b**) Differences of spectral phases between retrieved “Pulses II–V” and “Pulse I” (color curves) and theoretical estimations in parabolic approximation for corresponding dispersive compressor settings (black curves).

To demonstrate that the method proposed can be a powerful tool for real applications we have performed measurements for the single-shot implementation of the technique by using part of the high power laser system PEARL^34^. First of all, the laser system settings corresponding to minimal duration of experimentally measured SHIAC-function have been applied^35^. The pulse with full width at half maximum duration of 53 fs has been characterized. We have recorded the fundamental and two SPM-spectra after polyethylene terephthalate films^26^ with thicknesses of *L*_*1*_ = 0.75 mm and *L*_*2*_ = 1.5 mm in the single-shot mode. The left column in Fig. 6(a) (“Pulse I”) reflects these results for the estimated B-integrals of 0.8 after the first film and 1.6 after the second film consistent with the intensity of 0.3 TW/cm^2^. The algorithm has been run for the fundamental (first row) and two SPM spectra with the B-integrals differing about twice; see the second and third rows. One can see a good agreement between the retrieved and the original SPM spectra. The reconstructed pulse intensity distribution in the time domain is shown in the fourth row, and the numerically simulated for its SHIAC function ideally fitted to the experimentally measured one is shown in the fifth row. Next, the setup settings have been readjusted: the diffraction grating of the dispersive compressor^35^ has been shifted by certain distances relative to the optimal position in order to obtain pulses with changed parameters for examination. The changed pulses have been characterized using the proposed single-shot technique (see Fig. 6(a), “Pulse II–V”). In the first approximation, compressor detuning leads to additional parabolic spectral phases of pulses II–V (relative to pulse I). These phases (φ_k_ − φ_I_, k = II…V) have been theoretically estimated (see Fig. 6(b)). The developed retrieval method allows determining these contributions. The differences of the retrieved spectral phases of “Pulses II–V” and “Pulse I” are also shown in Fig. 6(b) by color curves which are very similar to the corresponding theoretically calculated black ones. It is a good confirmation of the method operability, particularly taking into account the almost perfect agreement between the measured SHIAC-functions and the numerically generated ones for reconstructed signals (see Fig. 6(a), 5^th^ row).

## Discussion and Conclusion

The presented numerical and experimental examples show the high applicability of the proposed method. One can see an ideal fit for unperturbed signals and very good retrieval results for the experimentally obtained single-shot pulses as well as the numerically generated signals with noise and many imperfections.

It should be noted that the method has no ambiguity related to time direction. It does not require a priori information about pulse shape and also allows estimating *B*-integrals which can be very useful for independent peak power evaluation. One can use, for example, quartz or plastic plates, such as polyethylene terephthalate^26^, with width of order 1 mm for intensities of order 1 TW/cm^2^. The main requirement is that dispersion effects should not significantly impact the pulse propagation through a thin plate. Note, however, that for correct operation of the algorithm, spectral broadening after a Kerr plate should be large enough. It usually corresponds to B-integrals of about 0.5–1 or higher. Otherwise, fundamental and SPM spectra are very similar and phase retrieval is ambiguous. The method is not sensitive to the absolute phase. The method has limitations related to resolution and range originating from the properties of the Fourier transform. Also, a measured pulse should not be highly chirped (it should be no more than about 3 times longer than a Fourier-limited one). Nevertheless, for highly chirped ultrashort signals, which are very challenging for FROG, SPIDER, SHIAC and other methods, the algorithm proposed here can be modified by searching spectral phase as a sum of quadratic and additional parts.

Thus, we have proposed a very simple method for ultrashort pulse reconstruction based only on fundamental and SPM spectra measurements after two thin nonlinear Kerr plates with B-integrals differing about twice. The Gerchberg–Saxton-like algorithm without back propagation procedure has been developed. It has been successfully used to demonstrate reconstruction of experimental and numerically generated ultrashort signals. The retrieved single-shot pulses from OPCPA laser system with parabolic phase difference between the pulses adjustable by a dispersive compressor are in a very good agreement with the corresponding SHIAC measurements. Numerical examples are provided for simple as well as for complex signals, including a double pulse and a few-cycle pulse with pedestal and dips down to zero spectral intensity.

This method can be used for TW and PW class laser systems operating in a single-shot mode in the optical, near- and mid-IR spectral ranges. The method is robust, low cost, stable to noise, does not require a priori information, and has no ambiguity related to time direction. It also allows estimating B-integrals, which may be very useful for independent peak power evaluation.

## Methods

Description of the proposed method and developed numerical algorithm is presented in the subsections “Description of the method” and “Numerical algorithm”, respectively.

### Experimental details

We have used the starting stage of the PW OPCPA laser complex PEARL^34^ producing laser pulses with minimal duration of about 50 fs, energy of 10–20 mJ and beam diameter of 20 mm. For single-shot registration of the fundamental and two SPM spectra, the laser beam has passed through the mask consisting of two flat parallel polyethylene terephthalate^26^ plates with thicknesses L_1_ = 0.75 mm and L_2_ = 1.5 mm, arranged one above the other with a small gap. After the mask, the laser pulse has been attenuated by ~100 times due to reflection from the front surface of the neutral density filter. Further, the pulse with decreased energy has been detected by image-spectrograph with a 2-dimensional sensor. One sensor coordinate corresponds to the signal wavelength, and the other corresponds to the position at the entrance slit of the image-spectrograph. So, spectra for various points in beam cross-section have been registered. In order to avoid spectra overlapping at the slit due to diffraction, the spectrograph has been placed as close as possible to the mask at a distance of a few centimeters from it. The neutral density filter has been used to inhibit a glint from the back face. The homogeneity of the intensity and temporal distribution of the tested beam area falling onto the slit of the spectrograph have been indirectly confirmed when we installed a plane-parallel plate of polyethylene terephthalate with thickness of 1.5 mm instead of a mask with further registration of homogeneous spectral modulation along the slit. We have also recorded SHIAC in the part of radiation branched from a thin (~200 μm) plane-parallel glass plate mounted in front of the mask. The estimations show insignificant plate impact on the analyzed pulse.

## Additional Information

**How to cite this article**: Anashkina, E. A. *et al*. Single-shot laser pulse reconstruction based on self-phase modulated spectra measurements. *Sci. Rep*. **6**, 33749; doi: 10.1038/srep33749 (2016).
